# Evaluating optical coherence tomography (OCT) findings as potential biomarkers in central nervous system (CNS) lymphoma with or without ocular involvement

**DOI:** 10.1186/s40942-021-00345-1

**Published:** 2021-11-24

**Authors:** Muhammad Hassan, Muhammad Sohail Halim, Rubbia Afridi, Nam V. Nguyen, Quan Dong Nguyen, Yasir J. Sepah

**Affiliations:** 1 Byers Eye Institute, Stanford University, 2370 Watson Court, Palo Alto, CA 94303 USA; 2 Ocular Imaging Research and Reading Center, Menlo Park, CA USA

## Abstract

**Background:**

To evaluate spectral domain optical coherence tomography (SD-OCT) findings as biomarkers in primary central nervous system lymphoma (PCNSL) with or without ocular involvement.

**Methods:**

This study was a cross-sectional study and patients with a confirmed diagnosis of PCNSL with or without ocular involvement were included. Patient cohort finder tool was used to identify patients with lymphoma using ICD-10 codes (C82–C88), from January 2004 to October 2017. A total of 14,820 patients were identified. Procedure code (92134) for optical coherence tomography (OCT) was then applied to identify patients who had underdone OCT imaging at ophthalmology clinic. Clinic charts of 460 patients with lymphoma and available OCT were reviewed to identify patients with confirmed diagnosis of PCNSL and divided into two groups (Group 1: with and Group 2: without ocular involvement). OCT scans of patients in both study groups were analyzed for the presence of (1) Hyperreflective deposits in choroid, retinal pigment epithelium (RPE), outer and inner retina; (2) RPE thickening; (3) Vitreous debris; (4) Intraretinal fluid; (5) Ellipsoid zone disruption by masked graders. Chi-square was used to analyze the difference between the groups.

**Results:**

Twenty-two eyes (11 patients) with PCNSL were included this study (Group 1: 6 eyes and Group 2: 16 eyes). Mean age of subjects was 65 years. Five patients (45.45%) were female. There was no statistically significant difference between the groups for the presence of hyperreflective deposits in choroid, RPE, outer and inner retina, and presence of RPE thickening, intraretinal fluid, and ellipsoid zone disruption. Vitreous debris was found more commonly in group 1 subjects (83%) than group 2 (31.25%) (p = 0.029). All subjects in both groups showed hyperreflective deposits in the RPE demonstrating RPE infiltration. However, RPE thickening was noted only in 3 patients (Group1: 1 and Group2: 2).

**Conclusions:**

OCT finding of hyperreflective deposits present in eyes with lymphoma secondary to PCNSL are also observed in eyes with PCNSL without ocular disease. However, the vitreous deposits are more commonly found in eyes with ocular disease. These hyperreflective deposits can serve as biomarkers for early detection of ocular involvement by PCNSL.

## Background

Intraocular lymphoma is a rare, aggressive, lymphocytic malignancy characterized by involvement of uveal tract, retina, optic nerve and vitreous cavity. The most common histological type is diffuse large B-cell lymphoma (DLBCL) with occasional T-cell origin [1, 2]. Intraocular lymphoma has been estimated to represent 1.86% of the ocular malignancies, 4–7% of the primary central nervous system (PCNS) lymphomas and 1–2% of the extra-nodal lymphomas [3].

Even though intraocular lymphoma is considered as a subtype of PCNSL, it can present independent of the central nervous system (CNS) involvement and labeled as primary vitreoretinal lymphoma (PVRL) [4, 5]. Only 15–25% of the PCNSL cases develop ocular involvement compared to 56–90% of the PVRL patients who eventually progress to involve the CNS [5, 6].

PVRL commonly presents as a diagnostic challenge to the ophthalmology community. It generally presents in elderly patients as chronic uveitic masquerade syndrome, which may initially respond to immunosuppressive therapy making it further challenging to identify the underlying etiology [7–9]. The gold standard for the diagnosis of the PVRL is identification of the lymphoma cells in ocular specimen, which is commonly achieved by histological and cytological evaluation of vitreous fluid after diagnostic vitrectomy or by choroidal/retinal biopsy [10].

Due to the invasive nature of the diagnostic procedures for intraocular lymphoma, it is important to identify potential non-invasive ocular imaging biomarkers that can be used to identify and monitor patients with intraocular lymphoma as a primary disease or with associated underlying PCNSL. One such biomarker is the presence of hyperreflective deposits on the spectral domain optical coherence tomography (SD-OCT) scans of the PVRL patients as identified by our group and others previously [11–14]. However, we do not know if these hyperreflective deposits are only limited to cases with PVRL or are also present in cases of PCNSL with or without ocular involvement in which case they may serve as biomarkers for potential future ocular involvement.

In this study, we aim to compare the SD-OCT scans of the subjects with PCNSL with and without ocular involvement for the presence of potential optical biomarkers that can in the future: (1) assist in the diagnosis and monitoring of response to therapy of PCNSL with ocular disease; (2) Play a role in early identification of subjects with PCNSL who are at risk of developing ocular involvement.

## Methods

The index study employed a retrospective cross-sectional design. All the subjects were selected from Stanford University Hospital who fulfilled the study inclusion and exclusion criteria. It was conducted in compliance with the Declaration of Helsinki, the United States Code of Federal Regulations Title 21, and the Harmonized Tripartite Guidelines for Good Clinical Practice (1996). The study was approved by local institutional review board. An informed consent waiver was obtained as charts of the selected patients were reviewed retrospectively.

### Inclusion and exclusion criteria

Subjects were included in the study if they fulfilled the following criteria: (1) Confirmed (by results of biopsy identified in the patient charts or by documented notes indicated that previous biopsy was performed and was positive) diagnosis of PCNSL irrespective of ocular involvement. (2) Had a high-quality macular region SD-OCT imaging scan performed within 1 month of the diagnosis of PCNSL. Subjects were excluded from the study if: (1) Had visual loss or retinal changes secondary to any ocular disease other than intraocular lymphoma; (2) Had a diagnosis of any malignancy other than PCNSL.

### Subject identification

The patient cohort finder tool was used to identify the patients with any type of lymphoma in the Stanford Hospital database using the ICD-10 codes (C82–C88), since January 2004 (when ICD codes were formally applied). Once the subjects with diagnosis of any type of lymphoma were identified, those who had an SD-OCT scan performed at the eye clinic (based on the procedure code (92134), were selected for further review. A retrospective review of clinic charts of this subgroups of subjects was performed to identify the subjects with a confirmed (by results of biopsy identified in the patient charts or by documented notes indicated that previous biopsy was performed and was positive) diagnosis of PCNSL. Furthermore, SD-OCT database of the ophthalmology unit was used to narrow the search to the subjects with diagnosis of PCNSL who had an SD-OCT scan of the macular region available within 1 month of the confirmed diagnosis of PCNSL. The subjects with confirmed diagnosis of PCNSL and with available SD-OCT scans within a month of diagnosis were further classified into those with ocular involvement (Group 1) and without ocular involvement (Group 2).

### Spectral domain optical coherence tomography (SD-OCT) evaluation

SD-OCT scans of the macular region captured using Heidelberg Spectralis (Heidelberg Engineering, Heidelberg, Germany) or Cirrus 5000 HD OCT (Carl Zeiss AG, Oberkochen, Germany) within one month of the confirmed diagnosis of PCNSL with or without ocular involvement were included in the study. The SD-OCT scans of these subjects were color inverted to black on white background within the original machine software to enhance the hyperreflective deposits within different layers. These scans were analyzed for the presence of: (1) Hyperreflective deposits in choroid, retinal pigment epithelium (RPE), outer retina and inner retina; (2) RPE thickening; (3) Vitreous debris; (4) Intraretinal fluid; (5) Ellipsoid zone disruption. These evaluations were performed by masked graders (MH, YJS). Any discrepancies were resolved with mutual discussion about the case. The hyperreflective deposits can appear as dots, nodules and bands as reported by our group previously [11].

### Outcome measures

Percentage of patients with presence of the following characteristics on the SD-OCT scans in both study groups: (1) Hyperreflective deposits in choroid, retinal pigment epithelium (RPE), outer retina and inner retina; (2) RPE thickening; (3) Vitreous debris; (4) Intraretinal fluid; (5) Ellipsoid zone disruption. Comparison of these ocular SD-OCT characteristics was also performed between the two study groups to document any similarities or differences. For the anatomical outcomes assessed on the SD-OCT, both eyes of the patients were included in the analysis individually as lymphoma could potentially affect each eye individually with different severities.

### Statistical analysis

Stata V14.1 (Stata Corp, TX, USA) was used to all statistical analysis. Chi-square was utilized to compare both study groups for presence of ocular characteristics of interest in both study groups. A p-value of < 0.05 was deemed significant.

## Results

### Subject identification

A total of 14,820 cases of all kinds of lymphomas were identified using the ICD-10 codes (C82–C88) on the cohort finder tool in the Stanford Hospital database from January 2004 to October 2017. Out of these patients which included all kinds of lymphomas, 460 subjects had undergone SD-OCT imaging at the Ophthalmology Clinic/Byers Eye Institute. The patient charts of these 460 subjects with all kinds of lymphomas were carefully reviewed to identify the subjects with biopsy proven diagnosis of PCNSL. Among these subjects, 11 subjects who had an SD-OCT scan available within 1 month of diagnosis of PCNSL were included in the study.

### Patient characteristics

Mean age of the study subjects at the time of diagnosis of PCNSL was 65 years. Five (45.5%) of the subjects were female. Three subjects (Six eyes) had had confirmed intraocular lymphoma diagnosis secondary to PCNSL and were classified into Group 1, while 8 subjects (16 eyes) had no ocular involvement and were classified into Group 2.

### Spectral domain optical coherence tomography characteristics

**Fig. 1:**
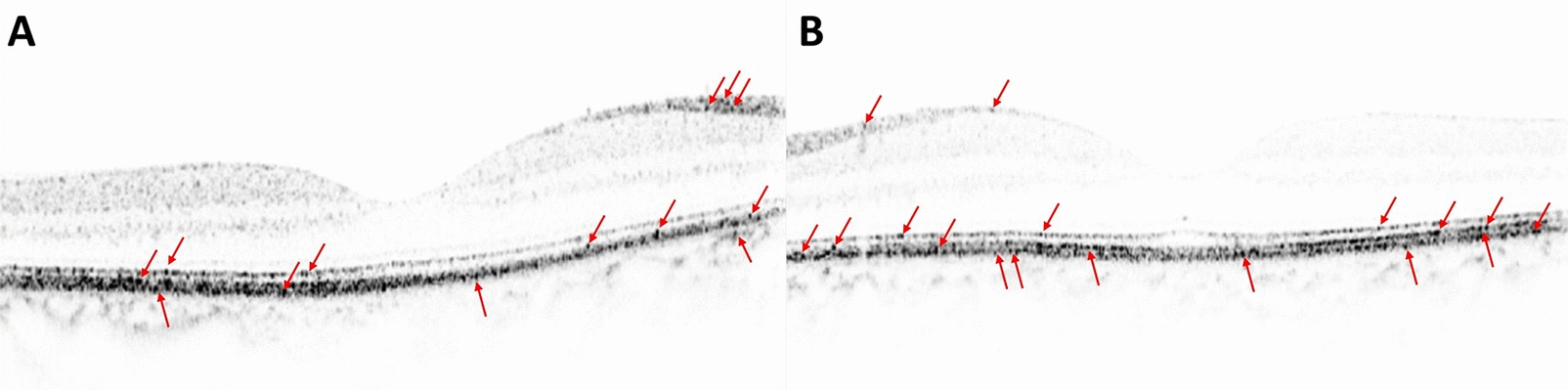
Spectral domain Optical Coherence Tomography (SD-OCT) scans of the eye with intraocular lymphoma (**A**) and without intraocular lymphoma (**B**) showing hyperreflective deposits in the retina (red arrows)

Table 1 shows the comparison of the SD-OCT characteristics between the two study groups For the purpose of these anatomical analysis, both eyes of the patients were included in the analysis individually. There was no statistically significant difference between the groups for the presence of hyperreflective deposits in choroid, RPE, outer and inner retina, and the presence of RPE thickening, intraretinal fluid and ellipsoid zone disruption (Fig. 1). Vitreous debris was found more commonly in group 1 subjects (83%) than group 2 (31.25%) and the difference between the two groups was significant (p = 0.029). All the subjects in both groups showed hyperreflective deposits in the RPE demonstrating RPE infiltration. However, RPE thickening was not observed in the majority of cases except in 3 patients (Group1: 1 and Group2: 2). Ellipsoid zone disruption was also seen more commonly in group 1 (50%) compared to group 2 (26.7%). However, the difference between the two groups was not statistically significant.

**Table 1:** Comparison of ocular characteristics between the two study groups

	Group 1 n (%)	Group 2 n (%)	p-value
Hyperreflective deposits—choroid	6 (100)	16 (100)	> 0.05
Hyperreflective deposits—retinal pigment epithelium	6 (100)	16 (100)	> 0.05
Hyperreflective deposits—outer retina	6 (100)	15 (93.7)	> 0.05
Hyperreflective deposits—inner retina	6 (100)	16 (100)	> 0.05
Retinal pigment epithelium thickening	1 (16.7)	2 (13.3)	> 0.05
Vitreous debris	5 (83.3)	5 (31.3)	*< 0.05*
Intraretinal fluid	1 (18.2)	3 (18.8)	> 0.05
Ellipsoid zone disruption	3 (50)	4 (26.7)	> 0.05

Group 1 consists of eyes of patients with primary central nervous system lymphoma (PCNSL) with ocular involvement and Group 2 consists of eyes of patients with PCNSL without ocular involvement

Statistically significant difference (in italic)

## Discussion

Intraocular lymphoma irrespective of primary origin (CNS or eye) presents as a diagnostic challenge to the ophthalmology community and often requires invasive diagnostic procedures to confirm the diagnosis (10). Therefore, it is important to identify non-invasive biomarkers that can help us to diagnose and/or monitor response to treatment in such patients. Patients diagnosed with PCNSL are referred to the retina and uveitis services at ophthalmic centers for evaluation of their eyes regardless of the presence or absence of ocular signs and symptoms. The absence of overt clinical signs of ocular involvement (such as the lack of vitreous cells or debris), however, may make it challenging to justify invasive procedures such as diagnostic vitreous biopsies. On the other hand, it is important to identify patients that are at risk of the disease progressing to involve the eye. This is important as the eye can act as a reservoir of persistent disease activity in patients where intraocular involvement is missed in cases of PCNSL [15]. Intraocular treatment provided in cases of PCNSL has been shows to prolong disease control although with no effect on overall survival risk. Therefore, the patients at risk can be followed more closely for any signs of intraocular disease [15]. Noninvasive imaging provides an ideal avenue to explore for optical markers of disease activity in the retina and vitreous of patients with PCNSL and or PVRL. In the index study, we utilized SD-OCT scans to demonstrate that even in the eyes that are clinically quiescent, there is a pattern of optical markers that is nearly identical to eyes with biopsy proven PVRL and eyes with intraocular lymphoma secondary to PCNSL.

We noted the presence of hyperreflective deposits at the level of choroid, RPE, outer and inner retinal layers in all eyes with intraocular lymphoma secondary to PCNSL in this study. Our group has previously reported similar hyperreflective deposits at the level of RPE, inner and outer retinal layers in subjects with PVRL [11, 13]. Similar findings in the RPE and outer retina have also been reported in cases of PVRL by other investigators [12, 14]. However, Barry et al. found choroidal hyperreflective deposits only in eyes with primary choroidal lymphoma and not PVRL [12]. These hyperreflective deposits have also been shown to increase with disease progression and regress in response to treatment in patients with PVRL [11, 14].

To our knowledge, our study is the first to identify and compare the SD-OCT findings among patients with PCNSL without ocular involvement to those with ocular involvement. We noted that all eyes of subjects with PCNSL without ocular disease demonstrated hyperreflective deposits at the level of choroid, RPE, outer and inner retinal layers similar to those seen in eyes with ocular disease secondary to PCNSL. Therefore, the results of our current study and the findings in the literature strengthen the hypothesis that PVRL with or without CNS involvement and PCNSL with or without ocular involvements lie on the same clinical-pathological disease spectrum [2, 10].

We did notice differences in two characteristics on the SD-OCT scans of the two groups. There was presence of vitreous debris in 83.3% of cases with PCNSL with ocular disease compared to 31.3% of cases with PCNSL without ocular involvement. Similarly, ellipsoid disruption was found to be more common in eyes with PCNSL with ocular involvement (50%) compared to those with PCNSL but no ocular disease (26.7%). However, only the presence of the vitreous debris in group 1 was statistically significant. Multiple studies have previously documented presence of vitreous debris in cases of PVRL as well [12, 14, 16]. These vitreous opacities have also been shown to decrease in number in response to treatment in patients with PVRL [14].

Based on the findings of our study and what has been reported in the literature, we hypothesize that the hyperreflective deposits seen in the retinochoroidal layers of eyes with PVRL and PCNSL with ocular involvement can potentially serve as biomarkers and can aid in the diagnosis of suspected clinical cases and the monitoring of disease activity in these two clinical entities. Additionally, the presence of these hyperreflective deposits in eyes of patients with PCNSL, which have not involved the eye yet, underscores the importance of studying these potential biomarkers in more details. Whether these deposits in eyes of patients with PCNSL point towards future ocular involvements, especially if there is presence of vitreous debris, or it may underscore the fact that we are potentially underdiagnosing the cases of PCNSL with ocular involvement as the overt symptoms have not developed yet. These questions may be determined by future prospective study designs.

Over 30% (31.3%) of eyes with PCNSL in our study were without any ocular involvement, based on clinical assessments, but had vitreous debris present on the SDOCT. These eyes also had other OCT findings that were similar to those of eyes with intraocular lymphoma. The majority of eyes without clinical assessments to have intraocular lymphoma did not have vitreous debris. Vitreous debris is present in the majority of eyes with intraocular lymphoma. Hence, our study may suggest that patients who have PCNSL without previous findings to suggest presence of intraocular lymphoma, who are then found to have OCT findings of vitreous debris and hyperreflective deposits, may undergo further diagnostic evaluations such as diagnostic vitrectomy to confirm the presence of intraocular lymphoma, which may aid the overall plan of therapy.

Even though the hyperreflective deposits is a consistent finding in the eyes of patients with PVRL with or without CNS involvement and PCNSL with or without ocular involvement, the exact nature of these deposits still yet needs to be evaluated. The deposits may represent lymphoma cells as those demonstrated in murine model [16]. Vasconcelos-Santos et al. also identified clusters of lymphoma cells on chorioretinal biopsy of a patient who had large sub-RPE deposits [17]. Similarly, vitreous debris more commonly seen in eyes with lymphoma may also represent clusters of lymphoma cells along with the associated inflammatory cells [16, 18, 19]. However, such correlation can only be confirmed by histopathological analysis of these hyperreflective deposits.

As with any study, our study has its limitations. We employed a cross sectional study design. We could not employ a prospective design to correlate the disease course with evolution of these hyperreflective deposits. Currently, patients with PCNSL may get an ophthalmic evaluation at the time of diagnosis but do not regularly return to the ophthalmology clinic in the absence of ocular symptoms as was seen with the cases we identified. Additionally, we used images from two different SD-OCT systems that have different resolution that may affect the identifications of the hyperreflective deposits. Furthermore, we did not have access to detailed analyzed systemic imaging data like magnetic resonance imaging for these patients and could not correlate our SDOCT findings to systemic imaging modalities.

## Conclusion

High resolution imaging by SD-OCT can detect the presence of hyperreflective deposits in chorioretinal layers of eyes of patients with PCNSL with or without ocular involvement. The presence of these hyperreflective deposits across this spectrum of disease underscores the importance of them as potential biomarkers in diagnosis and/or response to treatment in cases with ocular involvements and as an aid among early detection tools for future ocular involvements by PCNSL. Furthermore, presence of these deposits in the vitreous points more towards active ocular disease and can potentially serve as biomarker for response to therapy. Further studies are needed to prospectively follow patients with PCNSL and study changes in these biomarkers. Therefore, we highly recommend that patients with PCNSL should be followed by regular SD-OCT scans irrespective of presence of ocular symptoms.

## Data Availability

The datasets used during the current study are available from the corresponding author on request.

## Abbreviations

CNS: Central nervous system
IOL: Intraocular lymphoma
PCNSL: Primary central nervous system lymphoma
PVRL: Primary vitreoretinal lymphoma
SD-OCT: Spectral domain optical coherence tomography
